# Survival benefit of coronary-artery bypass grafting accounted for deaths in those who remained untreated

**DOI:** 10.1186/1749-8090-3-47

**Published:** 2008-07-17

**Authors:** Boris G Sobolev, Guy Fradet, Robert Hayden, Lisa Kuramoto, Adrian R Levy, Mark J FitzGerald

**Affiliations:** 1 Department of Health Care and Epidemiology, The University of British Columbia, Canada; 2 Department of Surgery, The University of British Columbia, Canada; 3 Department of Surgery, Royal Columbian Hospital, New Westminster, Canada; 4 Centre for Clinical Epidemiology and Evaluation, Vancouver Coastal Health Research Institute, Canada

## Abstract

**Background:**

Currently there are no direct estimates of mortality reduction afforded by coronary-artery bypass grafting (CABG) that take into account the deaths among patients for whom coronary revascularization was indicated but who did not undergo the treatment. The objective of this analysis was to compare survival after the treatment decision between patients who underwent CABG and those who remained untreated.

**Methods:**

We used a population-based registry to identify patients with established coronary artery disease who were to undergo first-time isolated CABG. We measured the effect of surgical revascularization on survival after the treatment decision in two cohorts of patients categorized by symptoms, coronary anatomy, and left ventricular function.

**Results:**

One in 10 patients died during the five years after treatment decision. The hazard of death among patients who underwent CABG was 51 percent of that for the untreated group, the adjusted hazard ratio was 0.51 (95 percent confidence interval, 0.43 to 0.61). The effect was stronger when CABG was performed within the recommended time: adjusted hazard ratios were 0.43 (95 percent confidence interval, 0.35 to 0.53) and 0.58 (95 percent confidence interval, 0.48 to 0.70) for early and late intervention, respectively; chi-square for the difference between hazard ratios was 12.2 (P < 0.001).

**Conclusion:**

Estimates that account for patients who died before they could undergo a required CABG indicate a significant survival benefit of performing early surgical revascularization even for patients registered to undergo the operation on the non-urgent basis.

## Introduction

Randomized trials and observational studies have demonstrated survival benefits conferred by coronary-artery bypass grafting (CABG) [1]. The intervention has been shown to improve long-term survival in stable symptomatic patients with left main coronary disease, triple-vessel disease, or two-vessel disease with significant stenosis of the proximal left anterior descending coronary artery [2]. In practice, however, personal reasons, scheduling procedures, or surgical wait lists can delay CABG after decision to operate has been made [3]. Hannan et al. argued that estimates of survival benefits conferred by CABG should account for the total number of deaths including deaths resulting from delays for required revascularization [4]. We anticipate that such estimates can show the difference in the risk of death between patients who actually undergo required CABG and those who remain untreated after the decision to treat has been made. The purpose of this analysis was to compare survival after the treatment decision between patients who underwent CABG and those who remained untreated. We used observational data from a population-based registry of patients with established coronary artery disease for whom surgical revascularization was indicated and planned. That registry collects information about adverse events on wait lists during the pre-operative period. Therefore it allows us to compare prognosis for both alternatives: if CABG is performed or if the patient remains untreated. In our view, such estimates introduce a methodological innovation, whereby deaths among untreated patients are used to estimate the risk of death for the treated group if they had remained untreated.

## Methods

### Data sources

Data were obtained from the British Columbia Cardiac Registry (BCCR). This prospective database contains dates of registration, procedure, and withdrawal, along with disease severity and other risk factors, for all patients who have been registered to undergo CABG in any of the 4 tertiary hospitals that provide cardiac care to adult residents of British Columbia, Canada, since 1991 [5]. To identify hospital admission and discharge dates, coexisting conditions, and in-hospital deaths, we used patients' Provincial Health Number to link deterministically BCCR records to the British Columbia Linked Health Database Hospital Separations File [6]. Data on coexisting conditions were retrieved in the form of diagnoses reported in discharge abstracts created during the calendar year before the treatment decision [7]. To identify deaths that did not occur in a hospital, we linked the BCCR to the British Columbia Linked Health Database Deaths File [8]. The University of British Columbia Ethics Board approved the study.

### Participants and outcomes

Our inception cohort consisted of all adult British Columbia residents with established coronary artery disease and a recorded request from a cardiac surgeon to book an operating room for isolated CABG in one of the participating hospitals between January 1, 1991, and December 31, 2000. We restricted the study to patients who required treatment on a semi-urgent or non-urgent basis and who had not previously undergone CABG (see Table 1 for criteria). We excluded 54 records that contained either insufficient information or invalid data: the registration and procedure dates were identical (50) or there was no surgical report (4). We excluded an additional 220 records for which registration and admission date suggested that the procedure had been performed immediately (115) or the request for the operating room had been sent and subsequently withdrawn several times (105). The study cohort thus consisted of 8,220 patients registered to undergo first-time isolated CABG.

**Table 1:** Definition of study groups

Group	Target Time for Surgery	Anginal Symptoms, Coronary Anatomy, and Left Ventricular Function
Semi-urgent	Within 6 weeks	Patients with either persistent unstable angina or stable angina and extensive CAD* (left-main stenosis more than 50 percent, triple-vessel disease, or double-vessel disease with significant proximal left anterior descending stenosis and impaired left ventricular function)
		
Non-urgent	Within 12 weeks	Stable symptomatic patients with limited CAD (double-vessel disease with no lesion in the proximal left anterior descending artery and normal left ventricular function or single-vessel disease with significant stenosis of the proximal left anterior descending artery)

*CAD = coronary artery disease.

Target Time = Recommended time

The primary outcome was death from any cause after the decision for surgery had been made. The follow-up time was counted as the number of months from the decision to treat until death, which may occur before surgery, after surgery but during the same hospital admission [10], and after discharge from the hospital, or a censoring event, whichever came first. Censoring events included removal from wait list due to being operated elsewhere, five years after the treatment decision (due to data availability), or December 31, 2002. The date of the surgeon's request to book the operating room served as the date of the decision for surgery and registration on a wait list. In British Columbia, surgical wait lists hold patient names until the surgery can be scheduled [9]. Patients are removed from a wait list without surgery if they die, if they reconsider the decision to undergo surgery, if their condition deteriorates such that surgery is no longer possible, or if they undergo surgery elsewhere.

In a separate analysis, we also studied deaths that occurred after discharge from the hospital. The follow-up time for patients who underwent surgery and were alive at discharge was counted as the number of months from discharge to death or 5 years or December 31, 2002, whichever came first. We excluded an additional 66 records from the analysis of long-term post-operative survival because the discharge date was invalid or missing.

### Statistical analysis

The average mortality rate – that is, the total number of deaths divided by the total number of patient-months at risk – was calculated for the study groups. Patients contributed time at risk to the untreated group until treatment, after which they contributed remaining time at risk to the treated group [11]. To measure the treatment-related effect, we used Cox regression with a time-dependent indicator variable that changed from 0 to 1 after surgery [12]. The exponential of the regression coefficient for this variable gave the hazard ratio for treated patients relative to those who remained untreated, a value of less than 1 indicating a lower hazard resulting from having undergone CABG. Such an approach allowed us to represent changes in the group membership (treated vs. untreated) over the follow-up time. The calculation assumes the ratio of hazards between groups is constant, whereas the hazard of death may evolve over time in each group.

We also studied whether survival differed significantly among patients who waited for CABG longer than the recommended time (6 weeks for patients in the semi-urgent group and 12 weeks for those in the non-urgent group [9]), using a separate model with two time-dependent indicator variables for CABG performed before and after the recommended time. To test for differences in post-operative survival between patients who underwent CABG within and after the recommended time, we used Cox regression with fixed covariates [13]. The timing of surgery was an indicator variable, a value of 1 denoting CABG within the recommended time. Point estimates and confidence intervals for hazard ratios were calculated for each urgency group and for the entire cohort.

We used multivariate models to control for differences in patients' characteristics and significant confounders (summarized in Table 2). In particular, we entered two indicator variables for the following categories of co-existing medical conditions: congestive heart failure, diabetes mellitus, chronic obstructive pulmonary disease, cancer, or rheumatoid arthritis [14]; and other co-existing chronic conditions listed in a clinical comorbidity index that was adapted for administrative data [15]. Presentation without co-existing conditions was the reference category. When performing the Cox regression analysis we stratified by age and sex to avoid the proportionality assumption for these variables. To assess whether the estimated models were consistent with our data we used the likelihood ratio test. All analyses were conducted with SAS version 8.2.

**Table 2:** Characteristics of patients in the study cohort by access to surgery after registration for CABG (as number and percentages across each characteristic)

		Access to surgery, % in each category			
Characteristic	% of all patients (N = 8220)	Unplanned emergency (N = 266)	Elective within target time (N = 2524)	Elective after target time (N = 4526)	Remained untreated (N = 904)
Age					
< 50 yr	666 (8.1)	19 (2.9)	209 (31.4)	370 (55.6)	68 (10.2)
50–59 yr	1814 (22.1)	55 (3.0)	518 (28.6)	1054 (58.1)	187 (10.3)
60–69 yr	3125 (38.0)	104 (3.3)	962 (30.8)	1727 (55.3)	332 (10.6)
70–79 yr	2457 (29.9)	84 (3.4)	787 (32.0)	1307 (53.2)	279 (11.4)
≥ 80 yr	158 (1.9)	4 (2.5)	48 (30.4)	68 (43.0)	38 (24.1)
					
Sex					
Female	1453 (17.7)	62 (4.3)	440 (30.3)	771 (53.1)	180 (12.4)
Male	6767 (82.3)	204 (3.0)	2084 (30.8)	3755 (55.5)	724 (10.7)
					
Urgency group					
Semi-urgent	6333 (77.0)	207 (3.3)	1986 (31.4)	3599 (56.8)	541 (8.5)
Non-urgent	1887 (23.0)	59 (3.1)	538 (28.5)	927 (49.1)	363 (19.2)
					
Comorbidity					
Major conditions*	1756 (21.4)	68 (3.9)	564 (32.1)	897 (51.1)	227 (12.9)
Other conditions^†^	2113 (25.7)	79 (3.7)	735 (34.8)	1067 (50.5)	232 (11.0)
None	4351 (52.9)	119 (2.7)	1225 (28.2)	2562 (58.9)	445 (10.2)
					
Coronary anatomy					
Left main	983 (12.0)	26 (2.6)	389 (39.6)	493 (50.2)	75 (7.6)
Multi-vessel^‡^	6583 (80.1)	213 (3.2)	1932 (29.3)	3718 (56.5)	720 (10.9)
Limited^§^	654 (8.0)	27 (4.1)	203 (31.0)	315 (48.2)	109 (16.7)
					
Period					
1991–1992	1507 (18.3)	50 (3.3)	596 (39.5)	697 (46.3)	164 (10.9)
1993–1994	1716 (20.9)	51 (3.0)	654 (38.1)	833 (48.5)	178 (10.4)
1995–1996	1663 (20.2)	53 (3.2)	333 (20.0)	1090 (65.5)	187 (11.2)
1997–1998	1714 (20.9)	65 (3.8)	422 (24.6)	1021 (59.6)	206 (12.0)
1999–2000	1620 (19.7)	47 (2.9)	519 (32.0)	885 (54.6)	169 (10.4)
					
Hospital					
1	1706 (20.8)	65 (3.8)	616 (36.1)	812 (47.6)	213 (12.5)
2	2759 (33.6)	74 (2.7)	964 (34.9)	1473 (53.4)	248 (9.0)
3	2013 (24.5)	58 (2.9)	372 (18.5)	1230 (61.1)	353 (17.5)
4	1742 (21.2)	69 (4.0)	572 (32.8)	1011 (58.0)	90 (5.2)

*Congestive heart failure, diabetes mellitus, chronic obstructive pulmonary disease, rheumatoid arthritis, cancer.

†Peripheral vascular disease, cerebrovascular disease, dementia, peptic ulcer disease, hemiplegia, renal disease, or liver disease.

‡Three- or two-vessel disease with stenosis of the proximal left anterior descending artery.

§Two-vessel disease with no lesion in the proximal left anterior descending artery or 1-vessel disease with stenosis of the proximal left anterior descending artery.

Target Time = Recommended time

## Results

### Access to surgery

Table 2 shows the baseline characteristics of the study cohort by access to surgery. Of the 8,220 patients registered for first-time isolated CABG, 7,316 (89.0 percent) underwent surgery, and 904 (11.0 percent) remained untreated because they died while awaiting CABG (80 or 1.0 percent), withdrew from treatment because they reconsidered the decision to undergo surgery, their condition deteriorated such that surgery was no longer possible or for other reasons **(**716 or 8.7 percent), or were lost to follow-up before the operation (108 or 1.3 percent). The median time from registration to surgery was 12 weeks (interquartile range, 6 to 23 weeks). Of patients in the semi-urgent group, 1,986 (31.4 percent) underwent elective CABG within the recommended time, 3,599 (56.8 percent) underwent elective CABG but after the recommended time, and 207 (3.3 percent) underwent the procedure via unplanned emergency admission. Among those in the non-urgent group, 538 (28.5 percent) underwent elective CABG within the recommended time, 927 (49.1 percent) underwent elective CABG but after the recommended time, and 59 (3.1 percent) underwent the procedure via unplanned emergency admission.

### Survival after treatment decision

One in 10 patients died during the first five years after the treatment decision: 80 while awaiting surgery, 141 after withdrawal from surgical treatment, 97 post-operatively during the admission for CABG, and 551 after hospital discharge (Table 3). The mean follow-up time after the treatment decision was 4.3 (standard error 0.02) years in the semi-urgent group and 4.3 (0.03) years in the non-urgent group.

**Table 3:** Characteristics of patients in the study cohort by death after registration for CABG *(as number and percentages within each category for each characteristic)

Characteristic	On wait lists (N = 80)	After withdrawal (N = 141)^†^	During admission (N = 97)	After discharge (N = 551)^‡^
Age				
< 50 yr	5 (6.3)	5 (3.5)	2 (2.1)	15 (2.7)
50–59 yr	17 (21.3)	20 (14.2)	17 (17.5)	73 (13.2)
60–69 yr	29 (36.3)	38 (27.0)	35 (36.1)	196 (35.6)
70–79 yr	28 (35.0)	64 (45.4)	41 (42.3)	244 (44.3)
≥ 80 yr	1 (1.3)	14 (9.9)	2 (2.1)	23 (4.2)
				
Sex				
Female	7 (8.8)	35 (24.8)	25 (25.8)	95 (17.2)
Male	73 (91.3)	106 (75.2)	72 (74.2)	456 (82.8)
				
Urgency group				
Semi-urgent	54 (67.5)	98 (69.5)	86 (88.7)	456 (82.8)
Non-urgent	26 (32.5)	43 (30.5)	11 (11.3)	95 (17.2)
				
Comorbidity				
Major conditions^§^	26 (32.5)	53 (37.6)	32 (33.0)	176 (31.9)
Other conditions^||^	20 (25.0)	36 (25.5)	23 (23.7)	130 (23.6)
None	34 (42.5)	52 (36.9)	42 (43.3)	245 (44.5)
				
Coronary anatomy				
Left main	6 (7.5)	13 (9.2)	12 (12.4)	75 (13.6)
Multi-vessel^¶^	66 (82.5)	112 (79.4)	81 (83.5)	447 (81.1)
Limited**	8 (10.0)	16 (11.3)	4 (4.1)	29 (5.3)
				
Period				
1991–1992	13 (16.3)	22 (15.6)	26 (26.8)	145 (26.3)
1993–1994	23 (28.8)	27 (19.1)	14 (14.4)	114 (20.7)
1995–1996	18 (22.5)	39 (27.7)	22 (22.7)	131 (23.8)
1997–1998	17 (21.3)	33 (23.4)	21 (21.6)	113 (20.5)
1999–2000	9 (11.3)	20 (14.2)	14 (14.4)	48 (8.7)
				
Hospital				
1	11 (13.8)	46 (32.6)	8 (8.2)	108 (19.6)
2	26 (32.5)	38 (27.0)	51 (52.6)	188 (34.1)
3	35 (43.8)	43 (30.5)	15 (15.5)	121 (22.0)
4	8 (10.0)	14 (9.9)	23 (23.7)	134 (24.3)

*Death on wait lists means any pre-operative death while on the wait list for surgery; death after withdrawal means any pre-operative death after removal from the wait list without surgery; death during admission means any death after surgery during the same hospital admission; death after discharge means any death after hospital discharge.

^†^Within 5 years after registration date.

^‡^Within 5 years after hospital discharge date, excluding two patients with unknown dates.

^§^Congestive heart failure, diabetes mellitus, chronic obstructive pulmonary disease, rheumatoid arthritis, cancer.

^||^Peripheral vascular disease, cerebrovascular disease, dementia, peptic ulcer disease, hemiplegia, renal disease, or liver disease.

^¶^Three- or two-vessel disease with stenosis of the proximal left anterior descending artery.

**Two-vessel disease with no lesion in the proximal left anterior descending artery or one-vessel disease with stenosis of the proximal left anterior descending artery.

Surgical revascularization significantly reduced mortality in the study cohort. The observed mortality rate was 1.7 deaths per 1000 patient-months in the treated patients and 4.0 deaths per 1000 patient-months in the untreated patients. The mortality rate was 1.5 and 1.8 deaths per 1000 patient-months for early (within the recommended time) and late (longer than the recommended time) treatment, respectively.

Among those who underwent CABG, the hazard of death was 51 percent of that in the untreated group, the adjusted hazard ratio 0.51 (95 percent confidence interval, 0.43 to 0.61) (Table 4). The effect was stronger when CABG was performed within the recommended time: the adjusted hazard ratios were 0.43 (95 percent confidence interval, 0.35 to 0.53) and 0.58 (95 percent confidence interval, 0.48 to 0.70) for early and late treatment, respectively (Wald chi-square = 12.2, df = 1, P < 0.001). The effect sizes for early and late treatment and the difference between them remained unchanged when an additional variable for undergoing CABG through unplanned emergency admission was entered into the regression model. In all models listed in Table 4, patients in the semi-urgent group were at a higher hazard of death than those in the non-urgent group, the adjusted hazard ratio ranging from 1.25 (95 percent confidence interval, 1.04 to 1.50) to 1.27 (95 percent confidence interval, 1.05 to 1.53). The full model was consistent with the data (likelihood ratio test = 148.9, df = 10, P < 0.001).

**Table 4:** Hazard ratios (HRs) for death at any time after registration for CABG for treated patients relative to untreated patients and for patients who had surgery within target times relative to those who had surgery after target times

Time-dependent treatment effect*	Unadjusted HR (95% CI)	Adjusted HR(95% CI)^†^
Model I, Treatment indicator		
Treated vs Untreated	0.53 (0.45–0.63)	0.51 (0.43–0.61)
		
Model II, Treatment indicators by target time		
Early Surgery vs Untreated	0.46 (0.38–0.57)	0.43 (0.35–0.53)^‡^
Late Surgery vs Untreated	0.59 (0.49–0.71)	0.58 (0.48–0.70)^‡^
		
Model III, Treatment indicators by target time and admission type		
Early Surgery vs Untreated	0.46 (0.37–0.57)	0.42 (0.34–0.52)^§^
Late Surgery vs Untreated	0.58 (0.48–0.71)	0.58 (0.48–0.70)^§^
Unplanned Emergency vs Untreated	0.59 (0.39–0.90)	0.56 (0.37–0.86)

*Among 8,220 patients who were registered to undergo CABG.

^†^CI denotes confidence interval.

^‡^Difference between regression estimates is significant (Wald chi-square = 12.2, df = 1, P < 0.001).

^§^Difference between regression estimates is significant (Wald chi-square = 12.3, df = 1, P < 0.001).

Target Time = Recommended time.

The survival benefit differed between urgency groups. For semi-urgent patients the mortality rates in the treated and untreated groups (1.8 and 4.6 per 1000 patient-months) were higher than those for non-urgent patients (1.3 and 3.1 per 1000 patient-months). The rates were 1.6 and 1.9 deaths per 1000 patient-months in early and late treatment groups of semi-urgent patients, and 1.1 and 1.4 deaths per 1000 patient-months respectively of non-urgent patients.

Compared with untreated patients, the hazard of death for those who underwent CABG was 46 percent lower in the semi-urgent group and 55 percent lower in the non-urgent group, and the adjusted hazard ratios were 0.54 (95 percent confidence interval, 0.44 to 0.66) and 0.45 (95 percent confidence interval, 0.32 to 0.63), respectively (Table 5). In each group, patients waiting longer than the recommended time were less likely than those with a shorter delay to benefit from the treatment. However, when CABG was performed after the recommended time, the survival benefit appeared to be smaller in the semi-urgent than the non-urgent group (adjusted hazard ratios 0.61 [95 percent confidence interval, 0.49 to 0.76] and 0.51 [95 percent confidence interval, 0.35 to 0.74], respectively). Among patients in the non-urgent group, treatment delay resulting in unplanned emergency admission reduced the survival effect to a non-significant level (adjusted hazard ratio 0.73 [95 percent confidence interval, 0.33 to 1.63]). The regression models were consistent with the data in both the semi-urgent group (likelihood ratio test = 103.5, df = 9, P < 0.001) and the non-urgent group (likelihood ratio test = 53.8, df = 9, P < 0.001).

**Table 5:** Hazard Ratios for death at any time after registration for CABG for treated patients relative to untreated patients and for post-operative death for patients who had surgery within target times relative to those who had surgery after target times (figures in brackets are 95-percent confidence interval)

	Semi-urgent group	Non-urgent group
Overall survival, time-dependent treatment effect*		
No. of deaths/no. of patients		
Treated patients	517/5,792	95/1,524
Untreated patients	152/541	69/363
		
Model I, Treatment indicator		
Treated vs Untreated		
Unadjusted hazard ratio	0.53 (0.43–0.65)	0.42 (0.30–0.59)
Adjusted hazard ratio	0.54 (0.44–0.66)	0.45 (0.32–0.63)
		
Model II, Treatment by target time		
Early Surgery vs Untreated		
Unadjusted hazard ratio	0.47 (0.37–0.59)	0.35 (0.23–0.55)
Adjusted hazard ratio	0.45 (0.36–0.58)	0.36 (0.23–0.56)
Late Surgery vs Untreated		
Unadjusted hazard ratio	0.58 (0.47–0.73)	0.47 (0.32–0.68)
Adjusted hazard ratio	0.61 (0.49–0.76)	0.51 (0.35–0.74)
		
Model III, Treatment by target time and admission type		
Early Surgery vs Untreated		
Unadjusted hazard ratio	0.47 (0.37–0.60)	0.32 (0.20–0.51)
Adjusted hazard ratio	0.45 (0.36–0.58)	0.33 (0.20–0.53)
Late Surgery vs Untreated		
Unadjusted hazard ratio	0.58 (0.46–0.73)	0.46 (0.32–0.67)
Adjusted hazard ratio	0.61 (0.48–0.76)	0.51 (0.35–0.74)
Unplanned Emergency vs Untreated		
Unadjusted hazard ratio	0.51 (0.31–0.84)	0.80 (0.37–1.76)
Adjusted hazard ratio	0.52 (0.32–0.87)	0.73 (0.33–1.63)
		
Long-term post-operative survival^†^		
No. of deaths/follow-up yrs		
Early Surgery	153/8,409	31/2,121
Late Surgery	303/13,883	64/3,648
Unadjusted hazard ratio	0.85 (0.70–1.03)	0.84 (0.55–1.29)
Adjusted hazard ratio	0.79 (0.65–0.96)	0.86 (0.55–1.33)

*Among 8,220 patients who were registered to undergo CABG.

†Within five years after discharge date among 7,153 patients who underwent surgery and did not die in hospital, after removal of 66 patients with invalid discharge dates

Target Time = Recommended time

### Long-term post-operative survival

Among the 7,316 patients who underwent CABG, 97 (1.3 percent) died post-operatively during the same hospital admission and 7,219 (98.7 percent) survived surgery. Of those who was discharged alive 551 (7.6 percent) died within five years. The observed death rate was 1.7 deaths per 100 patient-years for those who underwent surgery within the recommended time (184 deaths over 10,530 patient-years) and 2.1 deaths per 100 patient-years for those whose surgery was delayed (367 deaths over 17,531 patient-years). After adjustment, those who underwent CABG within the recommended time showed a 20 percent lower hazard of post-operative death than patients whose surgery was delayed (hazard ratio 0.80 [95 percent confidence interval, 0.67 to 0.95]).

Delays in treatment had a different effect in each group (Table 5). Among patients in the semi-urgent group (456 deaths over 22,292 patient-years), those who waited longer than the recommended time were less likely than those with a shorter delay to survive beyond five years. The observed rate of post-operative death was 1.8 deaths (95 percent confidence interval, 1.5 to 2.1) per 100 patient-years for those who underwent CABG within the recommended time and 2.2 deaths (95 percent confidence interval, 1.9 to 2.4) per 100 patient-years for those whose surgery was delayed. After adjustment, those who underwent CABG within the recommended time had a 21 percent lower hazard of late post-operative death than those with delayed surgeries (hazard ratio 0.79 [95 percent confidence interval, 0.65 to 0.96]). Among patients in the non-urgent group (95 deaths over 5,769 patient-years), the observed death rate was 1.5 deaths (95 percent confidence interval, 0.9 to 2.0) per 100 patient-years in those who underwent CABG during the recommended time and 1.8 deaths (95 percent confidence interval, 1.3 to 2.2) per 100 patient-years in those whose surgery was delayed. After adjustment, the effect of early surgery was not significant (hazard ratio 0.86 [95 percent confidence interval, 0.55 to 1.33]).

## Discussion

In this cohort of 8,220 patients with angiographically-proven coronary artery disease and planned surgical revascularization, we compared survival between patients who underwent CABG and those who remained untreated after the decision to treat was made. We found that the hazard of death among patients who underwent CABG was 51 percent of that in the untreated group (adjusted hazard ratio 0.51 [95 percent confidence interval, 0.43 to 0.61]). The effect was stronger when patients underwent surgery shortly after the treatment decision (adjusted hazard ratios 0.43 [95 percent confidence interval, 0.35 to 0.53] and 0.58 [95 percent confidence interval, 0.48 to 0.70] for early and late intervention, respectively). Relative to untreated patients, the hazard of death for those who underwent CABG was 46 percent lower in the semi-urgent group and 55 percent lower in the non-urgent group (adjusted hazard ratios 0.54 [95 percent confidence interval, 0.44 to 0.66] and 0.45 [95 percent confidence interval, 0.32 to 0.63], respectively).

CABG is indicated to alleviate symptoms and to reduce the risk of death among patients who have limiting angina that persists despite optimal medical treatment and suitable coronary anatomy [1]. To fully evaluate the survival benefit of CABG, the hazard of death for patients who have undergone CABG should be compared with the hazard of death for those who have not undergone the procedure, considering the intervention as an intermediate event in the course of disease. Because the group membership (treated vs. untreated) cannot be defined at the beginning of follow-up, we accomplished this by using the Cox regression with a time-dependent indicator variable for CABG occurrences during the follow-up period after registration for the treatment. The hazard ratio for that variable gives the estimated survival benefit, which a patient for whom surgical revascularization is indicated can interpret as the reduction in the risk of death offered by undergoing CABG relative to remaining untreated. Contrasting post-operative and pre-operative mortality had two premises. The first was that excluding pre-operative deaths and losses to follow-up relating to a longer wait for treatment would imply that survival time begins at the time of the procedure, rather than at the time of the decision to treat [3]. The second was that measuring mortality reduction afforded by undergoing CABG requires an allowance for the possibility that the patient undergoes treatment and dies anyway.

In observational studies, death before CABG has been reported to occur in 0.4 to 1.3 percent of patients for whom it was felt that surgery could be safely delayed [16-18]. Expressed in terms of group phenomena [19], these percentages may be less relevant to the patient who is more concerned with the fact that treatment delay may result in otherwise avoidable deaths. The analysis presented in our paper, which concludes that undergoing CABG rather than remaining untreated will reduce the risk of death by half, is more relevant to patients because it compares the 5-year prognosis for both alternatives: if CABG is performed or if the patient remains untreated.

A meta-analysis of trials comparing CABG and medical therapy showed that five-year mortality was 39 percent lower among patients who underwent CABG [2], which is similar to our findings for the semi-urgent group. The more profound effect in the non-urgent group was due to a 25 percent higher mortality rate for the semi-urgent group independent of the intervention. However, the fact that we considered all deaths among patients who had never undergone CABG, including patients who became not healthy enough to withstand the procedure or who reconsidered the surgery for other reasons, might have affected our estimates.

Our study was observational and therefore required risk adjustment for potential differences between treated and untreated patients. The existing literature suggests that elderly patients are more likely to undergo revascularization as an urgent procedure [20], that a smaller coronary vessel diameter may account for the higher risk of adverse cardiovascular events among women [21], that co-existing medical conditions may delay open heart surgery [22], that post-operative survival depends on institutional constraints and individual care providers [3], and that changes in practice or supplementary funds may reduce time until surgery [9]. All of these factors were entered into multivariate regressions in our study. Still, we were unable to adjust for upgrades in urgency during the pre-operative period. Because increasing urgency reduced the time to surgery, those who underwent CABG after an urgency upgrade might have been more ill, which might have resulted in a downward bias in the survival effect. On the other hand, several studies have shown that, when compared with medical charts, comorbid conditions are underreported in administrative databases among persons discharged after cardiovascular procedures [7,23,24]. Thus delayed surgery may be attributable to other unmeasured factors, which might have resulted in a upward bias in the survival effect for those unfit the operation. The quality of information about decision and withdrawal dates is a concern in this analysis. Although we considered the date of the booking request as the date of the decision for surgery, no audit was conducted to verify the accuracy of the coding dates in the BCCR records. Also, because we used only the British Columbia Linked Health Database Deaths File, we did not have information about patients who died or underwent CABG in other provinces.

This study has provided estimates of mortality reduction afforded by surgical revascularization, taking account of patients who died before they could undergo a required CABG. In particular, we found a significant survival benefit for patients registered to undergo coronary revascularization on the non-urgent basis. The survival benefit of CABG was greater when patients underwent the operation within the recommended time, suggesting that some of the benefit was lost by the delay beyond the recommended time.

## Authors' contributions

BS conceived the study concept and design, participated in analysis and interpretation, and drafted the manuscript. GF participated in data acquisition and critically revised the manuscript. RH participated in data acquisition. LK performed statistical analysis and drafted the manuscript. AL participated in data acquisition. MF critically revised the manuscript and has been involved in drafting the manuscript. All authors read and approved the final manuscript.
